# Koebnerization in Vasculitis: An Unusual Clinical Observation

**DOI:** 10.7759/cureus.90439

**Published:** 2025-08-18

**Authors:** Demita Nambam, Ranjeeta Sapam, Gurumayum Chitralekha Devi

**Affiliations:** 1 Dermatology, Venereology, and Leprosy, Jawaharlal Nehru Institute of Medical Sciences, Imphal, IND

**Keywords:** drug induced vasculitis, isomorphic response, koebner’s phenomenon, leukocytoclastic vasculitis, small vessel vasculitis, trauma-induced lesions

## Abstract

Koebner’s phenomenon (KP) refers to the development of isomorphic lesions of an existing dermatosis in otherwise uninvolved skin following trauma. While this response is well-established in conditions like psoriasis, lichen planus, and vitiligo, its occurrence in cutaneous vasculitis is exceedingly rare, with few reported cases. The authors present a rare case of a 47-year-old female with drug-induced vasculitis showing koebnerization, highlighting trauma as a potential exacerbating factor in vasculitic dermatoses. Trauma-induced vascular inflammation, immune complex deposition, or local cytokine release may be implicated in this unusual presentation.

## Introduction

Koebner’s phenomenon (KP) refers to the emergence of skin lesions on previously normal skin following trauma, which appear both clinically and histologically similar to an existing dermatosis [1]. Heinrich Koebner, a German dermatologist, was the first to describe this isomorphic response in psoriasis patients [2]. This response is classically described in psoriasis, vitiligo, and lichen planus [3]. However, its occurrence in vasculitic conditions, especially cutaneous small vessel vasculitis (CSVV), is rare and sparsely documented in the literature. CSVV typically presents as palpable purpura on dependent areas and is often triggered by infections, drugs, or autoimmune conditions. We report a rare case of drug-induced CSVV in which trauma appeared to induce a Koebner response.

## Case presentation

A 47-year-old female presented to the outpatient department with multiple red palpable rashes associated with moderate itching, which started on the lower extremities and spread to involve the abdomen, chest, and upper limbs over the course of five days. Three days after scratching, discrete, reddish lesions appeared along the scratch line. Palms and soles were spared. There was an associated low-grade, intermittent fever preceding the appearance of the rash by one day. Upon inquiry, she had taken a few tablets of over-the-counter azithromycin and paracetamol to manage her fever, two days after which the rash appeared.

She denied any abdominal pain, arthralgias, photosensitivity, hematuria, or melena. There is a history of prior over-the-counter intake of azithromycin; however, the patient reports no history of allergic reaction to this drug. No other allergic reactions to any medications are known to her previously. No previous episodes or significant personal or family history of autoimmune or systemic illness was noted.

**Figure 1:**
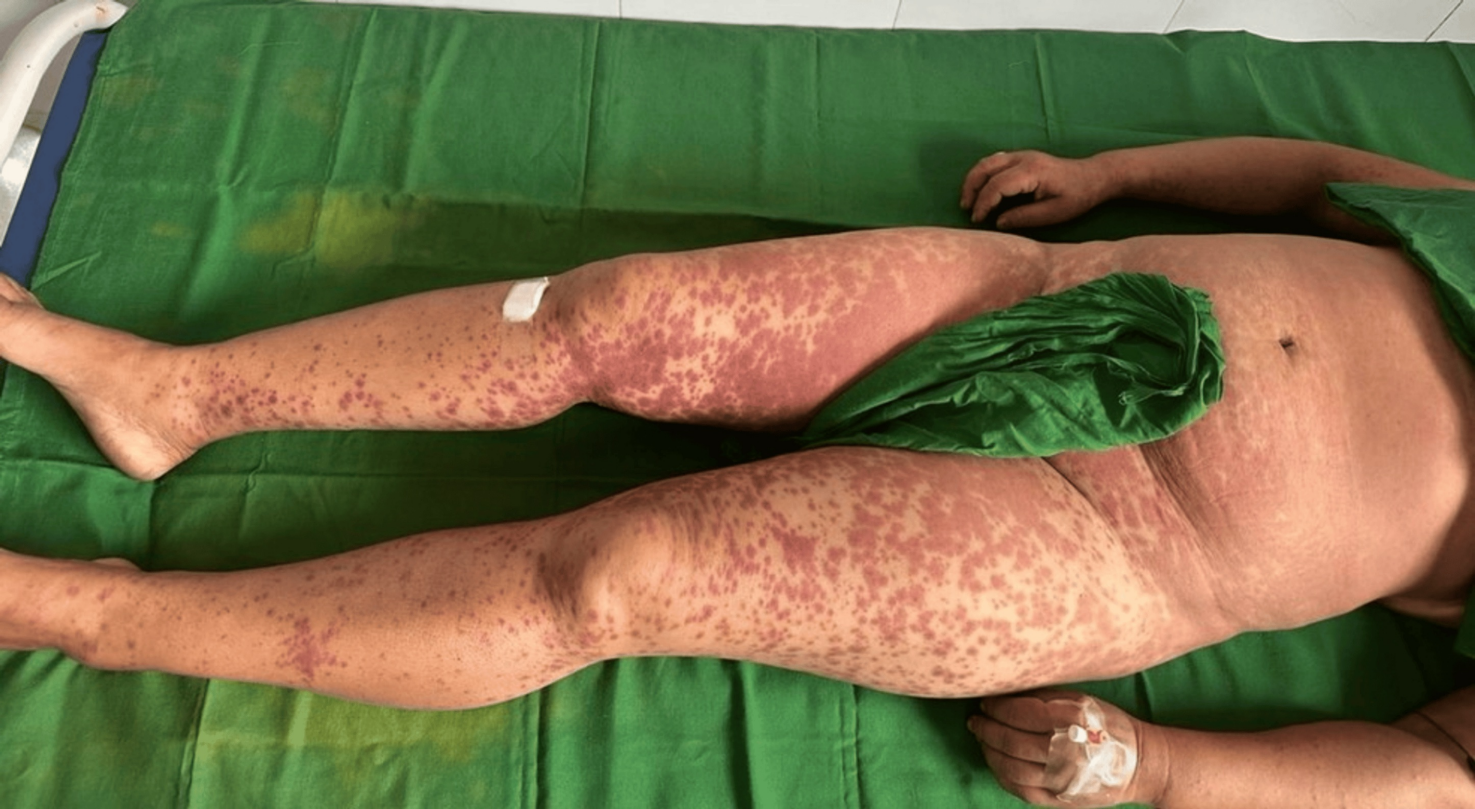
Discrete to coalescent, non-blanchable, purpuric papules to plaques distributed almost bilaterally symmetrically on the trunk and upper and lower extremities

**Figure 2:**
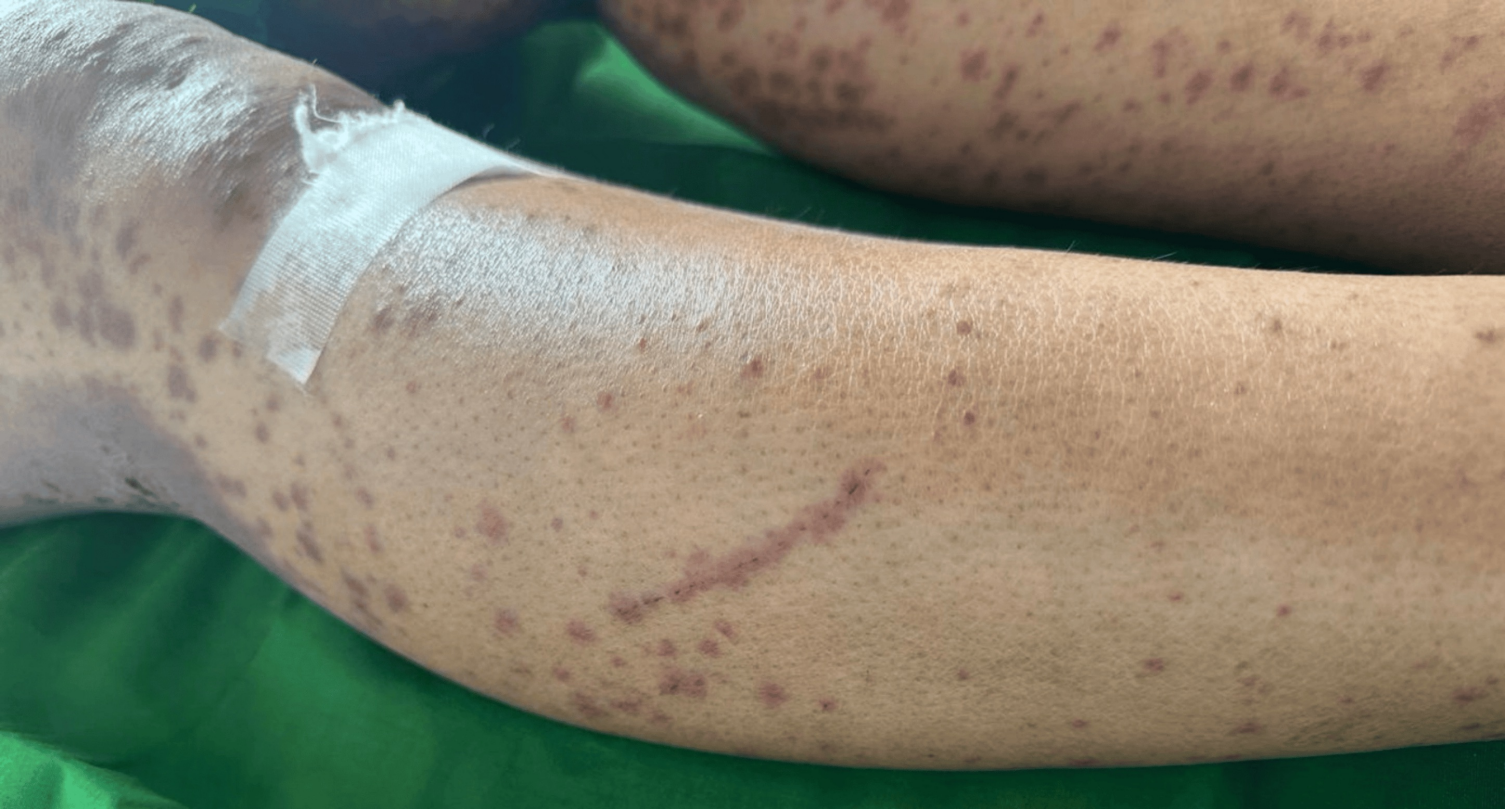
Purpuric papules in a linear configuration with excoriation

On mucocutaneous examination, multiple discrete to coalescent purpuric papules to plaques were noted on the chest, abdomen, and upper and lower extremities, almost bilaterally symmetrical (Figure 1). A striking linear arrangement of these similar purpuric papules was noted on the right shin with excoriation marks, which was consistent with KP (Figure 2). 

A biopsy taken from a lesion from the lower leg showed superficial perivascular lymphohistiocytic inflammatory infiltrate with few eosinophils, conspicuous nuclear debris, extensive extravasation of RBCs, and intravascular fibrin deposition. Direct immunofluorescence showed C3 deposits in papillary dermal vessels and was negative for IgA and IgG. Hence, a clinicopathological diagnosis of drug-induced vasculitis was made.

Serological workup showed an elevated erythrocyte sedimentation rate (ESR) and C-reactive protein (CRP). The rest were unremarkable, including normal antinuclear antibody (ANA), negative hepatitis B and C serology, and normal liver and kidney function tests (Table 1).

**Table 1:** Laboratory findings ESR: erythrocyte sedimentation rate; CRP: C-reactive protein; ANA: anti-nuclear antibody; LFT: liver function test; AST: aspartate aminotransferase; ALT: alanine aminotransferase; ALP: alkaline phosphatase; KFT: kidney function test; ELISA: enzyme-linked immunoassay

Tests	Value	Reference levels
ESR	50 mm/hour	0-20 mm/hour
CRP	2.4 mg/ml	>1 mg/ml
ANA (ELISA)	56	0-200 IU/ml
LFT		
Total Bilirubin	0.9	0.2-1.3 mg/dl
Direct Bilirubin	0.3	0.1-0.4 mg/dl
AST	38	15-46 U/L
ALT	42	13-69 U/L
ALP	87	38-126 U/L
Total Protein	8.1	6.3-8.2 gm/dl
Albumin	4.2	3.5-5.0 gm/dl
KFT		
Blood Urea	22	15-45 mg/dl
Creatinine	0.7	0.5-0.9 mg/dl

The patient was started on systemic corticosteroids at tablet prednisolone 40 mg and then tapered off gradually over two months, with significant improvement in symptoms. There was no report of relapse of symptoms on follow-up. Some lesions healed, leaving behind residual hyperpigmentation.

## Discussion

The KP is typically associated with preexisting skin disease, induced by either internal or external trauma. According to the classification by Boyd & Nelder, entities that commonly display KP, such as psoriasis, vitiligo, and lichen planus, belong to group 1, true koebnerization. It is reproducible in all patients by a variety of insults [4]. Leukocytoclastic vasculitis (LCV) is classified under group 4, questionable trauma-induced process, due to the paucity of reports. In our case, new eruptions were observed along the line of scratching, appearing three days after the initial trauma. The lesions progressed from discrete, reddish, palpable, non-blanchable papules to dusky discoloration and eventually healed with post-inflammatory hyperpigmentation within one week. If the lesions had been purely reactionary wheals, they would have appeared immediately after scratching and resolved without any change in color or residual pigmentation. Therefore, the delayed onset, morphological evolution, and healing pattern in this case are consistent with the KP.

LCV is a neutrophilic small vessel vasculitis resulting from the deposition of circulating immune complexes. Drug-induced vasculitis is an inflammation of blood vessels caused by the use of various pharmaceutical agents [5]. Drug-induced vasculitis accounts for a significant subset of CSVV, with common triggers including antibiotics and nonsteroidal anti-inflammatory drugs (NSAIDs) [5]. Azithromycin and paracetamol, although infrequent culprits, have been implicated in hypersensitivity reactions leading to vasculitis. Our patient had no systemic involvement or laboratory evidence of autoimmune disease, supporting the diagnosis of limited, drug-induced CSVV.

Although uncommon in LCV, KP has been described at sites of scars, excoriations, and areas subjected to pressure by tight clothing, with Henoch-Schonlein purpura being the most commonly described type [3]. In the case of CSVV, trauma-induced koebnerization may be explained by localized endothelial injury, immune complex deposition, and the release of pro-inflammatory cytokines; pressure-related microvascular stasis; mast cell activation; elevated tryptase levels; and vascular remodeling within scar tissue.

Kassam et al. [4] opined that the paucity of reports of KP in LCV may be attributed to the uncommon association of pruritus in LCV and predicted that there could be a rise in the cases reported if vasculitis was simultaneously pruritic from other causes, including other systemic illnesses. Therefore, they suggested consideration of the inclusion of LCV under true koebnerization, given the slow but steady increase of reported cases.

This is further supported by our observation of a linear arrangement of purpuric papules with overlying excoriation marks, consistent with trauma-induced lesion distribution.

## Conclusions

This case highlights an unusual and rarely reported manifestation of KP in drug-induced CSVV. The delayed, trauma-localized eruption observed here underscores the importance of meticulous history-taking and careful cutaneous examination to detect koebnerization, thereby revealing the patient’s susceptibility to disease propagation following minor trauma or scratching.

Recognizing mechanical injury as a potential precipitating factor in vasculitis not only aids in early diagnosis but also enables targeted patient counseling and preventive strategies such as the avoidance of scratching, friction, or other physical triggers during active disease phases.

From a therapeutic standpoint, early initiation of antihistamines may be beneficial, not merely for symptomatic pruritus relief, but also as a strategy to break the itch-scratch cycle and potentially halt trauma-induced lesion progression.

The distinctive presentation in this case, where a drug-induced vasculitic process exhibited a clear, localized koebnerization pattern adds a novel dimension to the clinical spectrum of CSVV. Further research is warranted to clarify the pathophysiological mechanisms linking mechanical trauma to vascular inflammation in such contexts, which may open avenues for both preventive and therapeutic interventions.
